# Pan-genome analysis of *Bacillus* for microbiome profiling

**DOI:** 10.1038/s41598-017-11385-9

**Published:** 2017-09-08

**Authors:** Yihwan Kim, InSong Koh, Mi Young Lim, Won-Hyong Chung, Mina Rho

**Affiliations:** 1 Department of Biomedical Informatics, Hanyang University, Seoul, Korea; 2 Department of Physiology, Hanyang University, Seoul, Korea; 3 Research Group of Gut Microbiome, Korea Food Research Institute, Seongnam, Gyeonggi-do Korea; 4 Department of Computer Science and Engineering, Hanyang University, Seoul, Korea

## Abstract

Recent advances in high-throughput sequencing technology allow for in-depth studies on microbial genomes and their communities. While multiple strains of the same species could display genomic variations with different gene contents in diverse habitats and hosts, the essential functions for a specific species are conserved as core genes that are shared among strains. We have comprehensively analyzed 238 strains of five different *Bacillus* species to identify the properties of core and strain-specific genes. Core and strain-specific genes in each *Bacillus* species show significant differences in their functions and genomic signatures. Using the core genes defined in this study, we have precisely identified the *Bacillus* species that exist in food microbiomes. Without resorting to culture-based whole genome sequencing, an unexpectedly large portion of the core genes, 98.22% of core genes in *B. amyloliquefaciens* and 97.77% of *B. subtilis*, were reconstructed from the microbiome. We have performed a pan-genome analysis on the core gene data of multiple *Bacillus* species to investigate the *Bacillus* species in food microbiome. Our findings provide a comprehensive genetic landscape of the *Bacillus* species, which is also consistent with previous studies on a limited number of strains and species. Analysis based on comprehensive core genes should thus serve as a powerful profiling tool to better understand major constituents in fermented food microbiomes.

## Introduction

Over the last decade, the number of sequenced bacterial genomes has doubled. Such data are also made available to the public to stimulate genomic research. Analysis on genomic data has revealed that multiple strains from diverse habitats and hosts show genomic variations in terms of gene contents. The essential functions for a specific species, however, are conserved as core genes that are shared among different strains. For example, *Streptococcus* species has been studied extensively for pan-genome analysis. As it turned out, 74% of *S. pneumonia* genome contents that was analyzed with 44 strains^1^, 80% of *S. agalactiae* with 8 strains^2^, and 75% of *S. pyogenes* with 11 strains^3^ are core genes. In addition, analysis on 44 strains of *S. pneumonia* has shown that the *Streptococcus* species shares 25% of genes in the same genus^1^.

With many strains of the same species becoming available, strain-specific functions are also attracting significant interests. Many clinical strains that are pathogenic or opportunistic have been sequenced, and investigated to identify clinically important genes and functions^4^. These studies have revealed that virulence factors and antibiotic resistance genes obtained from other organisms are encoded in strain-specific manner. Such horizontal transfer is an important mechanism by which microorganisms adapt to diverse environments for survival. Identification of strain-specific genes should thus advance our understanding of newly acquired functions of important microorganisms.

Ever since the concept of pan-genomes was first applied to the eight strains of *S. agalactiae* in 2005^2^, such analysis has been used to sharpen our understanding of bacterial genomic structures and diversity. Although the pan-genome could be an entire set of genes in any given taxon, it is mainly defined for the species, including all core genes, dispensable genes, and strain-specific genes^5–7^. The *core genes* are defined as the genes that exist in all strains. This implies that orthologous genes exist in all strains of a species, and usually perform essential functions for the specific species. If a gene from certain strain is separately clustered as a singleton, such gene is considered as a *strain-specific gene*. Strain-specific genes are sometimes called accessory genes. The *dispensable genes* are shared partially among strains. Overall, the number of core genes decreases as the number of genomes in a species increases, whereas the number of strain-specific genes constantly increases with the number of genomes^8^. In order to understand the essential functions for the species or laterally transferred functions in a specific strain, these different types of genes need to be investigated comprehensively.

Core, dispensable, and strain-specific genes are classified mostly based on the sequence homology among the genomes. Here, the threshold for the homology is critical. According to the classical definition, bacterial strains that show greater than 70% of DNA reassociation in the experiments are considered as a species^9^. Recent pan-genome analyses, on the other hand, consider the sequence similarity of genes of multiple strains to define bacterial species. In the pioneering research, Tettelin *et al*. applied sequence alignment algorithms with the minimum threshold of 50% sequence conservation^2^. Later studies have used more stringent thresholds of 70% similarity to find orthologous gene clusters^10, 11^.

Ever since the first *Bacillus* strain, *B. subtilis* 168, was sequenced in 1997^12^, *Bacillus* has become one of the most extensively studied species with the largest sets of genomes sequenced^13^. Analysis of the core genes and strain-specific genes in the *Bacillus* species, however, has been carried out in a very limited number of studies^14^. Many studies on *Bacillus* have focused on the pathogenic strains. For example, *B. anthracis* is responsible for the infectious disease anthrax, which affects humans and other animals^15, 16^. *B. cereus* is an opportunistic pathogen that is associated with food poisoning^15^. In order to better characterize the core genes of the species and across species, more comprehensive analysis needs to be applied on multiple strains and species of *Bacillus*. Additionally, it is critical to determine whether a specific species exists in the microbiome in order to analyze the bacterial community and their diversity.

In this study, we systematically investigated *Bacillus* genomes to identify the properties of core and strain-specific genes. Such core and strain-specific genes in each *Bacillus* species show significant differences in their functions and genomic signatures. Using the newly defined core genes, we have found *Bacillus* species in the fermented food microbiome. An unexpectedly large portion of core genes in *B. amyloliquefaciens* and *B. subtilis* were reconstructed from the microbiome without resorting to culture-based whole genome sequencing.

To the best of our knowledge, this is the first example of pan-genome analysis on multiple *Bacillus* species, and the application of core gene data to investigate the *Bacillus* species in food microbiome. Our findings provide a comprehensive genetic landscape of the *Bacillus* species, which is also consistent with previous studies on a limited number of strains and species. Moreover, a wider application of comprehensive core genes provided new tools to better understand the major constituents in the fermented food microbiome.

## Results

### Pan-genomes of the *Bacillus* species

Five *Bacillus* species were analyzed to understand the structures and functions of the pan-genomes. In particular, 20 strains of *B. amyloliquefaciens*, 52 strains of *B. anthracis*, 58 strain of *B. cereus*, 58 strains of *B. subtilis*, and 50 strains of *B. thuringiensis* were used to determine the core genes and strain-specific genes in the *Bacillus* pan-genomes (Table 1 and Supplementary Table S1). We only included almost complete genomes of the chromosome- and complete-level assembly in the NCBI repository to avoid incorrect implications from incomplete genomes that miss a set of genes in their assembly.

**Table 1:** Core and strain-specific genes in *Bacillus* species.

Bacillus species	# of strains	GC (%)	Avg genome size (Mbp)	Avg # of genes	Avg # of strain-specific genes	Avg # of core genes
*B. amyloliquefaciens*	20	46.37	3.96	3,842	68	2,870
*B. anthracis*	52	35.32	5.40	5,518	3	3,972
*B. cereus*	58	35.21	5.60	5,572	149	1,656
*B. subtilis*	58	43.90	4.04	4,031	52	1,022
*B. thuringiensis*	50	35.03	6.18	6,141	142	2,299

In order to find the core and strain-specific genes, we performed clustering on the entire set of protein sequences of each species, instead of using sequence comparison against a reference strain. Since different similarity thresholds have been used to find orthologous genes in previous studies^11^, we first clustered genes by using various thresholds of sequence similarity and length aligned to check the distribution of pairwise sequence similarities in each orthologous gene cluster. Notably, the average sequence similarity between the genes and the representative gene in each orthologous gene cluster was always above 96% when the threshold of sequence similarity ranged from 50% to 90% (Supplementary Table S2 and Supplementary Figure S1). Some dispensable genes showed relatively lower similarities between 86.66% and 99.77% depending on the threshold (Supplementary Table S2). With the 70% similarity threshold, the average similarity of orthologous genes in five *Bacillus* species were 97.14%, 99.62%, 93.23%, 94.71%, and 94.46% in *B. amyloliquefaciens, B. anthracis*, *B. cereus*, *B. subtilis*, and *B. thuringiensis*, respectively (Supplementary Table S3). This result strongly suggests that the similarity threshold for the clustering of orthologous genes does not significantly affect the clustering results, since orthologous genes within the same species are highly conserved. As a result, 70% of sequence similarity and 70% of aligned length of shorter sequence could be properly used in this study. This threshold have been used in the previous studies to cluster orthologous genes for comparative analysis^10, 11^.

Overall, core genes are well conserved in the *Bacillus* species. A total of 2870, 3972, 1656, 1022, and 2299 core gene clusters were determined in *B. amyloliquefaciens, B. anthracis*, *B. cereus*, *B. subtilis*, and *B. thuringiensis*, respectively (Table 1 and Supplementary Table S3). The proportion of the core genes corresponds to 75.20%, 74.01%, 30.87%, 26.01% and 38.75% of each gene contents, respectively. The average sequence similarity of the core genes was 98.03%, 99.67%, 95.70%, 95.60%, and 95.85% in *B. amyloliquefaciens*, *B. anthracis*, *B. cereus*, *B. subtilis*, and *B. thuringiensis*, respectively (Supplementary Table S3). Among the *Bacillus* species that were surveyed in this study, *B. anthracis* shows the highest sequence similarity of 99.67% for the core genes. This result indicates that *B. anthracis* has the most well-conserved core genes among the strains. As discussed in the previous study, *B. anthracis* strains are significantly homologous, which possibly results from a recent evolution of the species^17^.

A total of 1364, 156, 8617, 3013, and 7087 strain-specific genes were determined in *B. amyloliquefaciens*, *B. anthracis*, *B. cereus*, *B. subtilis*, and *B. thuringiensis*, respectively (Supplementary Table S3). While similar numbers of strains were analyzed for *B. subtilis, B. cereus*, and *B. thuringiensis*, the number of strain-specific genes in *B. subtilis* was significantly lower than those in the other two species: 3,013 genes in *B. subtilis* vs 8,617 and 7,087 in *B. cereus* and *B. thuringiensis*, respectively. The average number of strain-specific genes is 68, 3, 149, 52, and 142 in each strain of *B. amyloliquefaciens*, *B. anthracis*, *B. cereus*, *B. subtilis*, and *B. thuringiensis*, respectively.

Overall, the pan-genome analysis on each *Bacillus* species identified 6,656 orthologous genes in *B. amyloliquefaciens*, 6,135 in *B. anthracis*, 21,675 in *B. cereus*, 10,116 in *B. subtilis*, and 20,882 in *B. thuringiensis* (Supplementary Table S3). For *B. amyloliquefaciens*, the size of pan-genome increases slowly as the number of strains decreases in a cluster of orthologous genes (Supplementary Figure S2A). In addition, when we constructed the pan-genome with subsets of *B. amyloliquefaciens* strains, the same pattern was also observed. Overall, *B. cereus* and *B. thuringiensis* have rather high genetic diversity, whereas *B. amyloliquefaciens* and *B. subtilis* have low genetic diversity, with respect to the number of strain-specific genes. *B. anthracis* shows the lowest diversity among the strains (Supplementary Figure S2).

### Differentially enhanced functions in core and strain-specific genes

**Figure 1:**
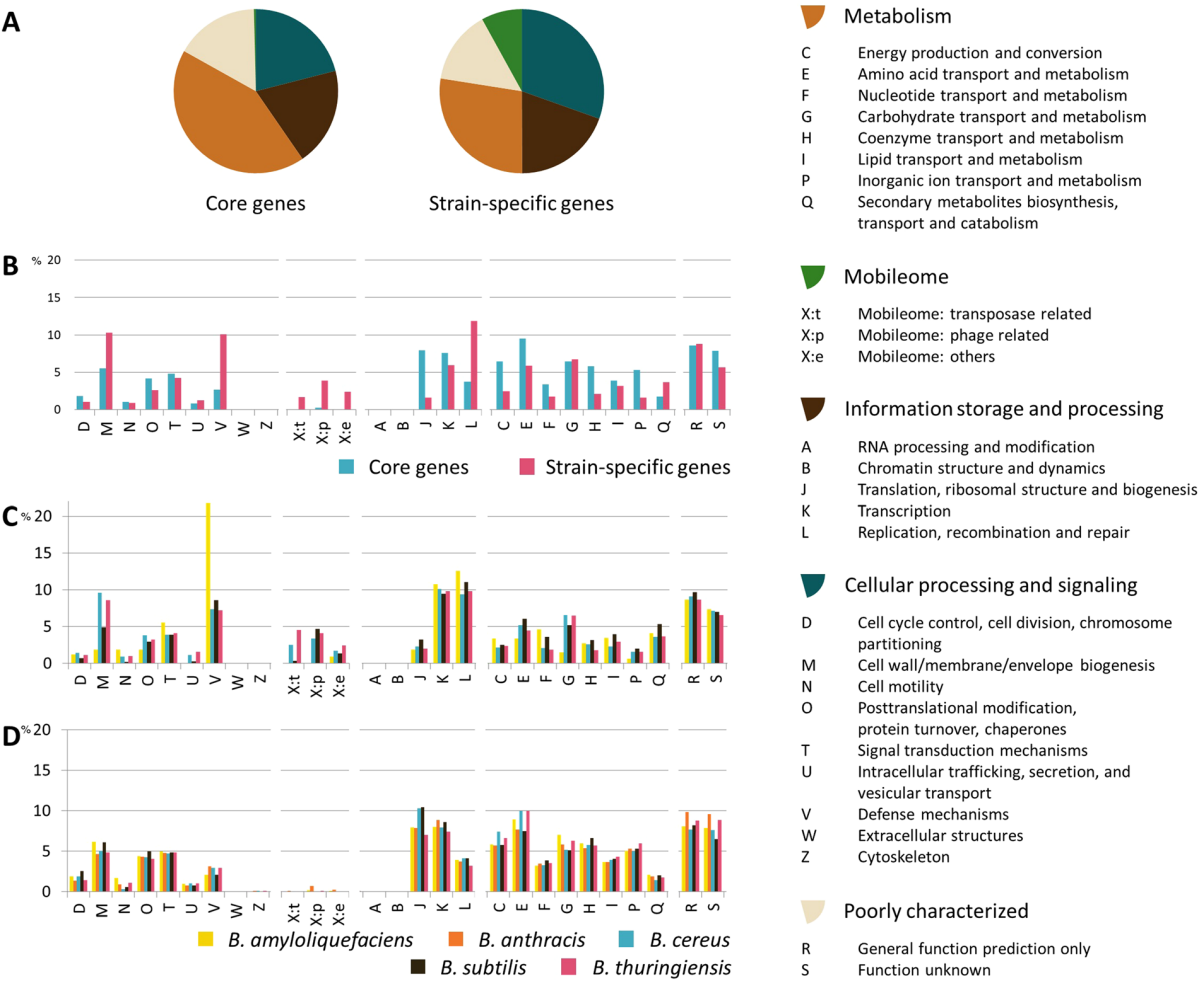
Differential distribution of COG functional categories in core and strain-specific genes: (**A**) Proportion of five classes of functional categories in core and strain-specific genes. (**B**) Functional categories in core and strain-specific genes. (**C**) Functional categories in the strain-specific genes of five *Bacillus* species. (**D**) Functional categories in the core genes of five *Bacillus* species.

Core and strain-specific genes were searched to compare the distribution of their functional categories by using the Clusters of Orthologous Groups of proteins (COGs) database^18^. The most abundant functions in the core genes of *Bacillus* species are associated with the metabolism (Fig. 1A). The overall proportion of metabolic functions in the core genes is 42.72%, whereas that in the strain-specific genes is 27.55%. The metabolism is almost 1.5 times more enhanced in the core genes, compared with the strain-specific genes. More specifically, energy production and conversion (C), amino acid transport and metabolism (E), coenzyme transport and metabolism (H), and Inorganic ion transport and metabolism (P) are noticeably more abundant in the core genes (p-value < 0.001) (Fig. 1B and Supplementary Table S7). This different proportion was similarly observed in all five *Bacillus* species (Fig. 1C and D).

The mobilome-related functions such as prophage and transposase proteins are significantly enhanced in strain-specific genes (Fig. 1A and Supplementary Table S7). While only less than 1% of the core genes are assigned to the mobilome-related functions, 0.92%, 7.57%, 6.40%, and 11.15% of strain-specific genes are assigned to such functions in *B. amyloliquefaciens*, *B. cereus*, *B. subtilis*, and *B. thuringiensis*. Our observation suggests that strain-specific genes might have been transferred horizontally from other species or even from other genus. A similar association was also observed in the previous study on *S. agalactiae* ^2^. In order to explore their functions in detail, we divided the mobilome category into two functions: transposase (i.e. X:t) and phage (i.e. X:p).

Interestingly, the functional category of information storage and processing shows highly different proportions in the sub-categories (Fig. 1B). The functions of translation, ribosomal structure, and biogenesis (J) are significantly enhanced (p-value < 0.001 in the core genes, whereas the functions of replication, recombination, and repair (L) are significantly enhanced (p-value < 0.0001 in the strain-specific genes (Supplementary Table S7). This feature might be due to the fact that replication, recombination, and repair are also highly involved in mobilome-related functions. This trend was also observed in previous studies^7^. In the cellular processing and signaling, the function of the defense mechanisms (V) is more abundant in the strain-specific genes (p-value < 0.05, compared with the core genes (Fig. 1B).

From the viewpoint of inter-species, the proportions of functional categories in the core genes are quite similar among the *Bacillus* species (Fig. 1D). Similar to the core genes, most of strain-specific genes show quite similar proportion of functions except a few. However, the function of defense mechanisms (V) is significantly more abundant in *B. amyloliquefaciens* (Fig. 1C). In addition, cell wall/membrane/envelope biogenesis (M) is abundant in *B. cereus* and *B. thuringiensis* as mentioned above.

### Different genomic signatures in core and strain-specific genes

**Figure 2:**
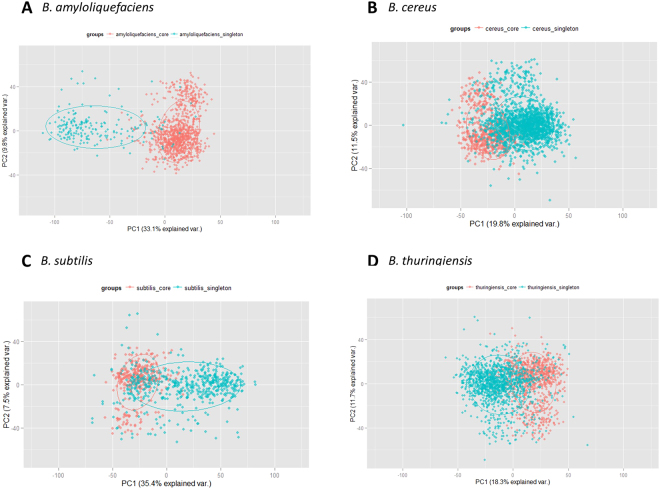
Comparison of the genomic signatures in core and strain-specific genes of *Bacillus* species.

*Bacillus* species has about 2.70%, 2.46%, 1.81%, 1.32%, and 0.06% of strain-specific genes for *B. cereus*, *B. thuringiensis*, *B. amyloliquefaciens*, *B. subtilis*, and *B. anthracis*, respectively. By using an interpolated Markov model, we inspected how the genome signature of strain-specific genes is different from that of the core genes. In the *Bacillus* species that we investigated, the genome signature of strain-specific genes is notably different from those of the core genes. Specifically, *B*. *amyloliquefaciens* and *B. subtilis* show significantly high variance in the first principle component in the PCA analysis (Fig. 2 and Supplementary Figure S3). The genomic signatures of core and strain-specific genes in *B. cereus* and *B. thuringiensis* are less distinctive compares to those in *B*. *amyloliquefaciens* and *B. subtilis*, but still show the difference for the first principle component. This finding could imply that strain-specific genes might have been transferred from other genomes.

### Phylogenetic analyses

**Figure 3:**
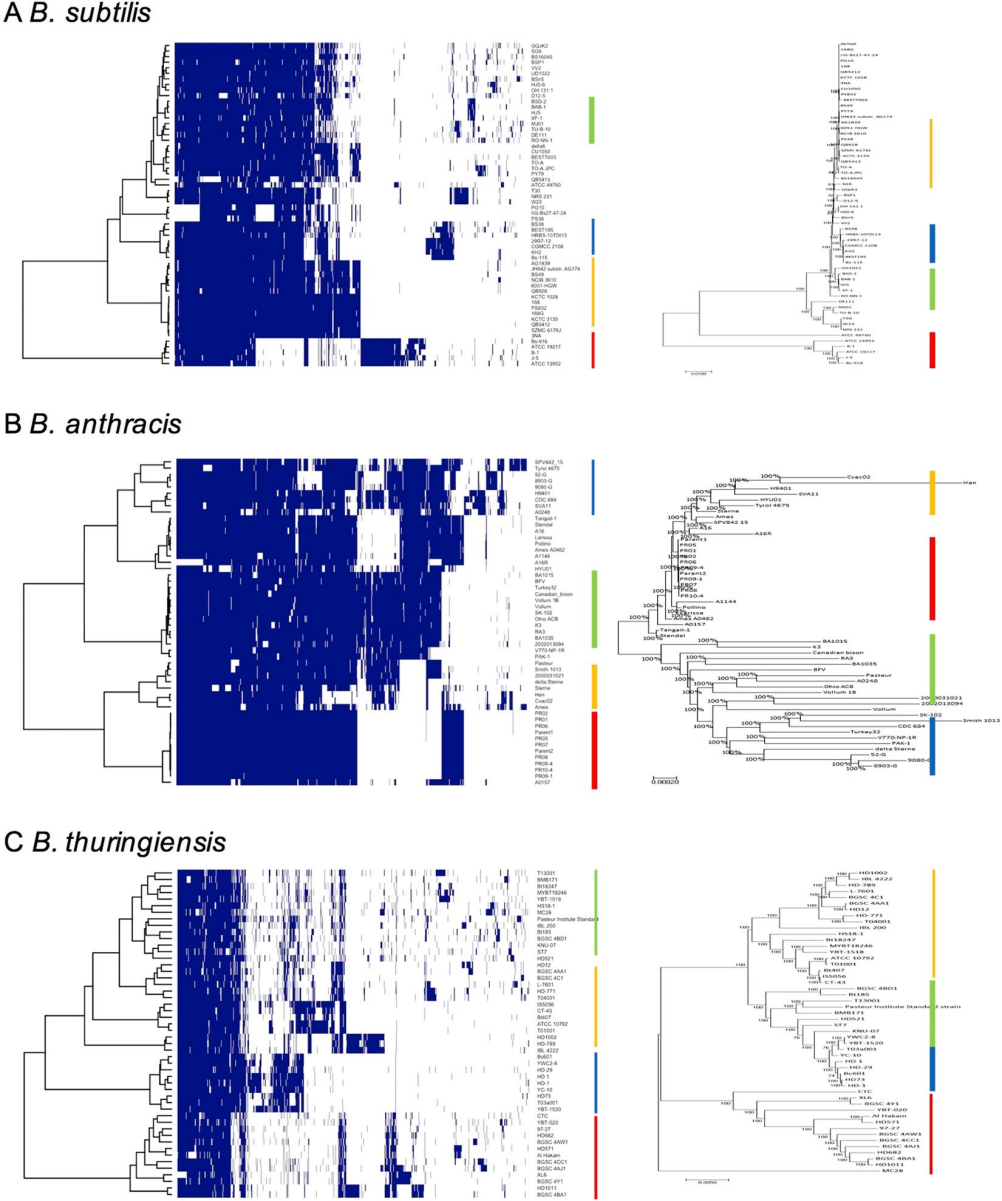
Clustered heatmap based on the shared genes (left) and phylogenetic tree of core genes (right). The strains grouped with the core genes in the phylogenetic tree (right) correlate with the clusters of the dispensable genes (left) in *B. subtilis* (**A**), *B. anthracis* (**B**), and *B. thuringiensis* (**C**).

The phylogenetic relationships among the strains in five *Bacillus* species were investigated by using two different approaches (Fig. 3). The first approach is building phylogenetic trees with the core genes aligned. Multiple sequence alignments were performed for each core genes in each species, and combined all together to build the trees. The other approach is using the proportion of dispensable genes between strains. This approach is also expected to find the evolutionary relationship among strains since the strains that share more genes have been observed in the close lineages. Average proportions of shared genes in each species are 61.86%, 82.02%, 71.63%, 79.39%, and 63.86% in *B. amyloliquefaciens, B. anthracis, B. cereus, B. subtilis*, and *B. thuringiensis*, respectively.

**Figure 4:**
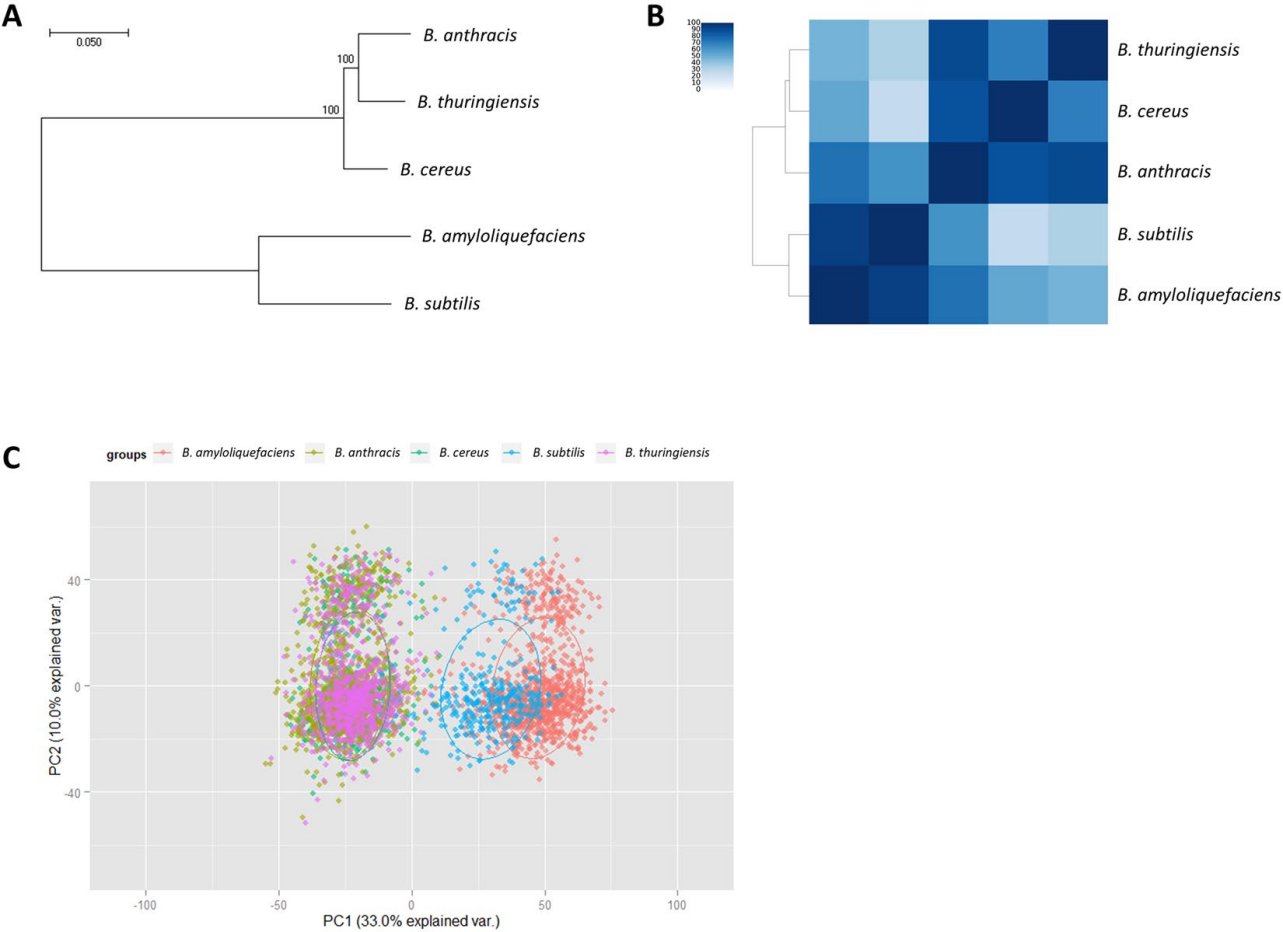
Phylogenetic tree of five *Bacillus* species. (**A**) Phylogenetic tree of the core genes shared by all five species. (**B**) Clustering of *Bacillus* species based on the proportion of the shared genes between species. (**C**) PCA analysis on the genomic signature of the core genes of the five *Bacillus* species.

We also compared the core genes in each *Bacillus* species with each other to find the conservation among species. In the phylogenetic tree that was built on the core genes from multiple species (i.e. *Bacillus* core genes), we observed that the species are divided into two groups (Fig. 4). One group is mainly human-host species such as *B. thuringiensis*, *B. cereus*, and *B. anthracis*. The other group is mainly environment-habitat species such as *B. subtilis* and *B. amyloliquefaciens*. The clustering with respect to the number of shared genes showed the same results as the phylogenetic tree with core genes (Fig. 4A and B). In addition, such two groups of *Bacillus* species show significantly different genome signature (Fig. 4C).

### *Bacillus* in the *doenjang* microbiome

Profiling a specific species and genus is an important procedure to understand microbiome. Currently, several computational methods are applied to profile taxonomy of the microbiome^19^. Many methods, however, rely on one or more marker genes to estimate the proportion of taxa in the microbiome. In this analysis, we instead performed more comprehensive reconstruction of core genes, and applied it to find precisely the *Bacillus* species existing in food microbiomes. For each *Bacillus* species from fermented food microbiomes, our study reliably confirmed which *Bacillus* species exist and how diverse their strains are.

*Doenjang* is a traditional Korean fermented soybean paste, which is similar to miso in Japan and tempeh in Indonesia^20^. Naturally transferred microorganisms are involved in the fermentation process, and influence the profile of many nutritious components in *doenjang* ^21^. In order to profile *Bacillus* species in the five *doenjang* microbiomes, the entire set of *Bacillus* core genes were clustered with the genes predicted from the *doenjang* microbiomes.

Clustering was conducted with two different similarity thresholds: 70% and 90%. With 70% similarity threshold to find core genes of each species, we observed that on averages, 99.29%, 99.71%, 20.63%, 27.45%, and 18.16% of the core genes in *B. amyloliquefaciens*, *B. subtilis, B. thuringiensis, B. cereus*, and *B. anthracis* respectively, were clustered with the genes in each microbiome (Supplementary Table S4). This result strongly suggests that *B. amyloliquefaciens* and *B. subtilis* exist as major components in the *doenjang* microbiome, which is consistent with the marker gene-based taxonomy profiling (Supplementary Table S5). On the other hand, among the core genes in *B. thuringiensis*, *B. cereus*, and *B. anthracis* that were clustered with the genes predicted from the microbiome, about 20.68% are *Bacillus*-specific core genes that are shared by all five species.

**Figure 5:**
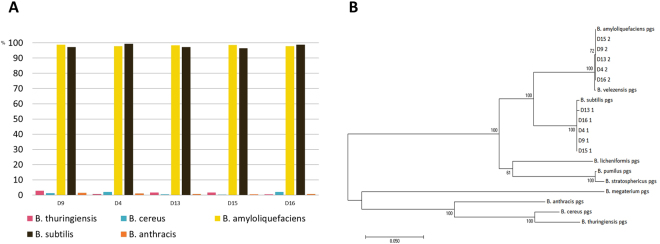
Identification of *Bacillus* species in the *doenjang* microbiome. (**A**) The ratio of clustered core genes with genes in *doenjang* microbiome of each species. Phylogenetic trees of three *Bacillus* genes of (**B**) *pgs*ABC genes in five microbiomes D4, D9, D13, D15, and D16, along with the genes in the reference genomes.

In order to further confirm this observation, we performed another clustering with more stringent threshold of 90% of sequence similarity (Supplementary Table S4). As we observed in the analysis of core genes in *Bacillus* species, the average pairwise similarity between orthologous genes of strains was above 93%. Such stringent threshold resulted in a better clustering among different *Bacillus* species. On average, 98.22%, 97.77%, 1.30%, 0.83%, and 0.86% of the core genes in *B. amyloliquefaciens*, *B. subtilis, B. thuringiensis*, *B. cereus*, and *B. anthracis* were exclusively clustered with the genes in five microbiomes (Fig. 5A and Supplementary Table S4).

In order to find phylogenetic relationships between the *Bacillus* strains in the *doenjang* microbiome and known strains, a gene complex, poly-γ-glutamate (PGA), was used. PGA is a fermentation product, and is composed of glutamate residues with γ-amino bonds^22^. Three PGA genes, *pgsA, pgsB*, and *pgsC*, in five microbiomes D4, D9, D13, D15, and D16, were aligned with the genes in the *Bacillus* reference genomes (Fig. 5B and Supplementary Figure S4). The phylogenetic tree reveals that the *Bacillus* strains in the microbiome are strongly associated with two *Bacillus* species groups of *B. amyloliquefaciens* and *B. subtilis*, as observed in the core gene-based analysis of *Bacillus* composition in the microbiome. Even though we did not include other *Bacillus* strains such as *B. velezensis* in our comparative analysis due to the limited number of complete genomes, they were included in this analysis for a better resolution of phylogenetic tree. These species are phylogenetically close enough to *B. amyloliquefaciens* and *B. subtilis*, and have been observed in other food microbiomes as well. In addition, other *Bacillus* species such as *B. anthracis* and *B. cereus* are separately branched in the tree, implying that the *Bacillus* core genes could be used for a better resolution of *Bacillus* composition in the *doenjang* microbiome.

## Discussion

In this study, we investigated *Bacillus* genomes to reveal the properties of core and strain-specific genes. Core and strain-specific genes in each *Bacillus* species show significant differences in genomic signatures, which is the evidence of lateral gene transfer. Our functional analysis on core and strain-specific genes also showed that many strain-specific genes are related with transposase and prophage. Based on this observation, we suggest that core genes of each species be used as a tool to identify *Bacillus* species in the microbiome.

Using the core genes newly defined in this study, we have identified *Bacillus* species with a high resolution in the fermented food microbiome. An unexpectedly large portion of the core genes in *B. amyloliquefaciens* and *B. subtilis* were reconstructed from the microbiome without resorting to culture-based whole genome sequencing. In addition, phylogenetic analysis with the core genes from the microbiome and annotated genes from reference genomes strongly suggests that all five *doenjang* microbiome contains *B. amyloliquefaciens* and *B. subtilis* species.

There still exist certain limitations in using core genes to identify bacterial species. First, the amount of sequenced genomes is not sufficient for diverse species. While we had to use only five species among diverse *Bacillus* species in this study, our method could be generally applicable and readily extendable to other species when their genomes become available. In addition, our core gene-based identification method, in conjunction with the phylogenetic analysis, can perform the taxonomic assignment with high resolution. Second, the appropriate similarity threshold value might be different from species to species. While the value of 95% used in this work seems to be generally applicable for *Bacillus* species, the similarity threshold value for other species needs to be carefully selected and evaluated since the evolution rate is different for different species.

## Methods

### Identification of core genes and strain-specific genes in *Bacillus* species

A total of five *Bacillus* species, *B. amyloliquefaciens*, *B. anthracis*, *B. cereus*, *B. subtilis*, and *B. thuringiensis* were selected for the pan-genome analysis. Other *Bacillus* species have only a few strains with the complete genomes in NCBI repository (2017 June), and are thus excluded in this study in order to avoid incorrect implications. A detailed genomic information on the strains of these five species are summarized in Supplementary Table S1.

In order to identify orthologous genes among the strains in *Bacillus* species, CD-HIT^23^ was applied to cluster all the genes in each species. Based on CD-HIT clustering algorithm, the sequence similarity threshold is defined as the similarity between the center sequence (i.e. representative sequence) in each orthologous gene cluster and a sequence. The amino acid sequences of the genic regions were used. CD-HIT clustered all the genes with the thresholds of 70% for protein sequence similarity, and 70% for shorter sequence alignment length^24^. In order to find reliable thresholds for clustering, we evaluated the clustering results with various ranges of sequence similarity and alignment length (Supplementary Figure S1).

If genes from all the strains in a *Bacillus* species are clustered in a group, such genes are considered as *core genes*. In other word, the species-core genes are shared by all the strains in a species. If a gene exists only in a cluster, it is considered as a *strain-specific gene* (singleton genes) in this study. If genes from some strains in a species are clustered in a group, they are considered as *dispensable genes*. For each cluster of various size, average pairwise-similarity between the representative gene and each gene in the cluster was calculated (Supplementary Figure S1).

For *Bacillus* genus pan-genome analysis, we obtained core genes of each species. A total of 2,870 clusters of orthologous genes in *B. amyloliquefaciens*, 3,972 in *B. anthracis*, 1,656 in *B. cereus*, 1,022 in *B. subtilis*, and 2,299 in *B. thuringiensis* were clustered together to find genus-specific core genes. CD-HIT^23^ clustered species-specific core genes with the threshold of 40% for sequence similarity, and 40% for shorter sequence alignment length.

### Characterization of strain-specific genes

In order to find the different nucleotide composition in core genes and strain-specific genes, we performed a principal component analysis (PCA) with 2^nd^-order Markov chains as features. Markov chain is the conditional probability that a certain nucleotide (A, C, G, or T) occurs right after a given sequence of nucleotides^25^. According to the length of such sequence, the order of Markov chain is defined. The 2^nd^-order MC means the probability that a nucleotide occurs when two specific nucleotides are given. In particular, we applied an interpolated Markov model^25^ to generate more efficient features even in shorter genes with a limited number of string patterns. When the frequency of 2^nd^-order Markov chain is less than 40 bps, 1^st^-order Markov chain was used. Such features were calculated for each gene. The results of PCA were visualized using the ggbiplot2 package in R.

### Evolutionary Analysis

In order to investigate the evolutionary relationship among strains, two methods were applied: multilocus sequence tags (MLST) and absence/existence pattern of dispensable genes. For MLST, multiple sequence alignment was performed separately for each core gene by using MUSCLE^26^. Subsequently, the multiple alignments were concatenated to produce one multiple alignment of the entire core genes in each species. The neighbor-joining phylogenetic tree was built for each species by using MEGA (ver. 7.0.14)^27^. For constructing the phylogenetic tree, the following options were used: Poisson model for substitution model; uniform rates among sites; complete deletion option for gaps/missing data; 100 replicates of the bootstrap test.

In the second approach of using the absence/existence pattern, the pattern of shared genes obtained from the pan-genome were calculated for each pair of strains. In the clustering using the average linkage, the pattern was used to find how each pairs of strains share genes in each species.

### Identification of functional categories for core and strain-specific genes

COG database^18^ was used to find the function of core and strain-specific genes. For each gene, five best hits against the COG database were obtained by using BLASTp. If more than three hits were assigned to the same COG ID, such COG ID was assigned to the gene. Otherwise, the genes were categorized as “uncharacterized”.

Each COG was assigned to one of the 26 functional categories. If more than one functional classes were assigned to a COG ID from five best hits, each function was assigned with equal partial weights of each functional category. For example, since COG0028 was assigned to EH, which is associated with the E category (amino acid transport and metabolism) and H category (coenzyme transport and metabolism), the weights for E and H categories were 0.5 each.

### *Doenjang* metagenome sequencing

Five *Doenjang* samples (D4, D9, D13, D15, and D16) were collected from five different local companies that produce *Doenjang* by traditional methods. Total DNA was extracted from each *Doenjang* sample, as previously described^28^, and stored at −20 °C prior to analysis. All samples were sequenced using the Illumina Hiseq. 2500 instrument according to the manufacturer’s instructions (Supplementary Table S6).

### Profiling *Bacillus* species in the microbiome

In order to profile the *Bacillus* distribution, MetaPhlAn^29^ was applied with default options (Supplementary Table S5). The shotgun metagenome reads were assembled by using MEGAHIT^30^ with default *k*-mer options. Subsequently, genes were predicted from the contigs by using FragGeneScan^31^. The genes longer than 500 bps were retained and clustered with the *Bacillus* core genes by using CD-HIT^23^. Two different similarity thresholds of 70% and 90% were applied to compare the clustering results. Since clustering with 70% similarity threshold has the possibility of including core genes at higher level taxon, more stringent clustering with 90% similarity threshold was also performed. The clustering resulted in the profiling of core genes for the major constituent of *Bacillus* species, *B. amyloliquefaciens* and *B. subtilis*, in the *doenjang* microbiome. In addition, the phylogenetic tree on *pgs*ABC genes in the microbiome was built along with the genes of the *Bacillus* species (Supplementary Figure S4). Multiple alignment was conducted with the *pgs*ABC genes from the reference genomes and the microbiomes by ClustalW^32^. The neighbor-joining phylogenetic tree was built by using MEGA (ver. 7.0.14). For constructing the phylogenetic tree, the following options were used: Poisson model for substitution model; uniform rates among sites; use all site option for gaps/missing data; 100 replicates of the bootstrap test.

### Data availability

All raw sequencing data described in this study is available at European Nucleotide Archive (ENA) with the accession number ERP021911.

## Electronic supplementary material

Supplementary info
