# Supplementary material for: Pan-genome analysis of Bacillus for microbiome profiling
Source: Sci Rep. 2017 Sep 8;7:10984. doi: 10.1038/s41598-017-11385-9 (PMC5591210; doi:10.1038/s41598-017-11385-9)
Supplement: Supplementary file 1 — Supplementary info [file 41598_2017_11385_MOESM1_ESM.pdf]

Pan-genome analysis of *Bacillus* for microbiome profiling

Yihwan Kim[1,+], InSong Koh[1,2,+], Mi Young Lim[3], Won-Hyong Chung[3], and Mina Rho [1,4*]

[1]Department of Biomedical Informatics, Hanyang University, Seoul, Korea

[2]Department of Physiology, Hanyang University, Seoul, Korea

[3]Research Group of Gut Microbiome, Korea Food Research Institute, Seongnam, Gyeonggi-do, Korea

[4]Department of Computer Science and Engineering, Hanyang University, Seoul, Korea

[+] Equal Contributors

*Correspondence: minarho@hanyang.ac.kr

Supplementary Figure Legend

Supplementary Figure S1. Number of the core genes (top) and their average similarities in the orthologous gene cluster (bottom). Clustering thresholds ranges from 50% to 90% in different color.

Supplementary Figure S2. Pan-genomes in five *Bacillus* species. (A) Total number of orthologous gene clusters with respect to the number of strains in a cluster. (B) Sequence similarity distribution of genes in each cluster of *B. amyloliquefaciens* with respect to the number of strains in a cluster.

Supplementary Figure S3. Comparison of the genomic signatures (containing PC3) in core and strain-specific genes of *Bacillus* species.

Supplementary Figure S4. Identification of *Bacillus* species in the *doenjang* microbiome. Phylogenetic trees of three *Bacillus* genes of (A) *pgs*A, (B) *pgs*B, (C) *pgs*C genes in five microbiomes D4, D9, D13, D15, and D16, along with the genes in the reference genomes.

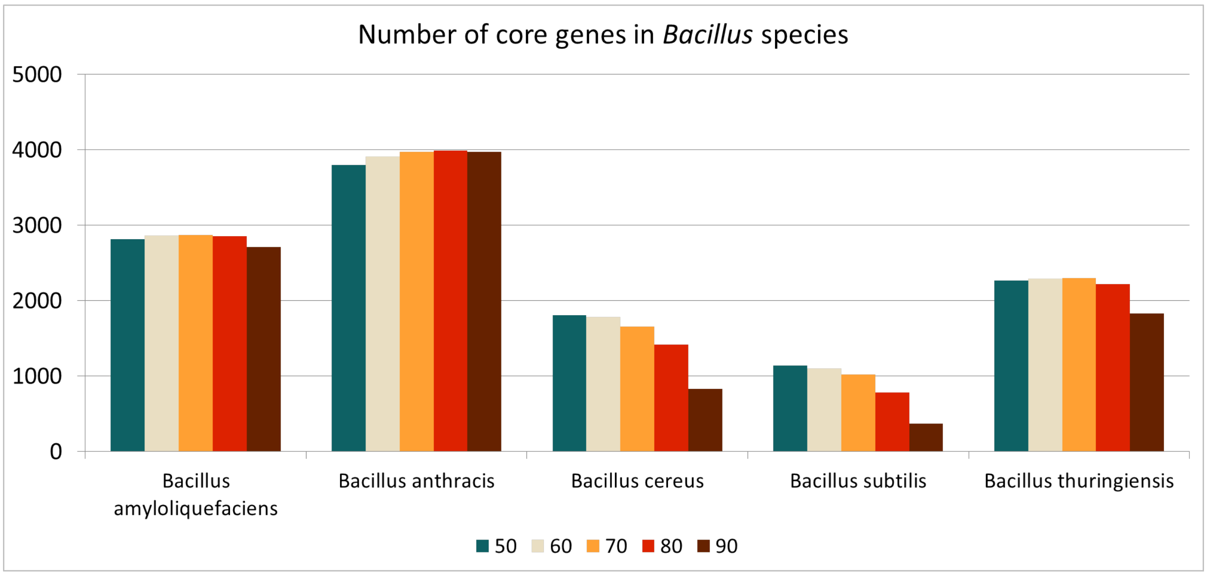

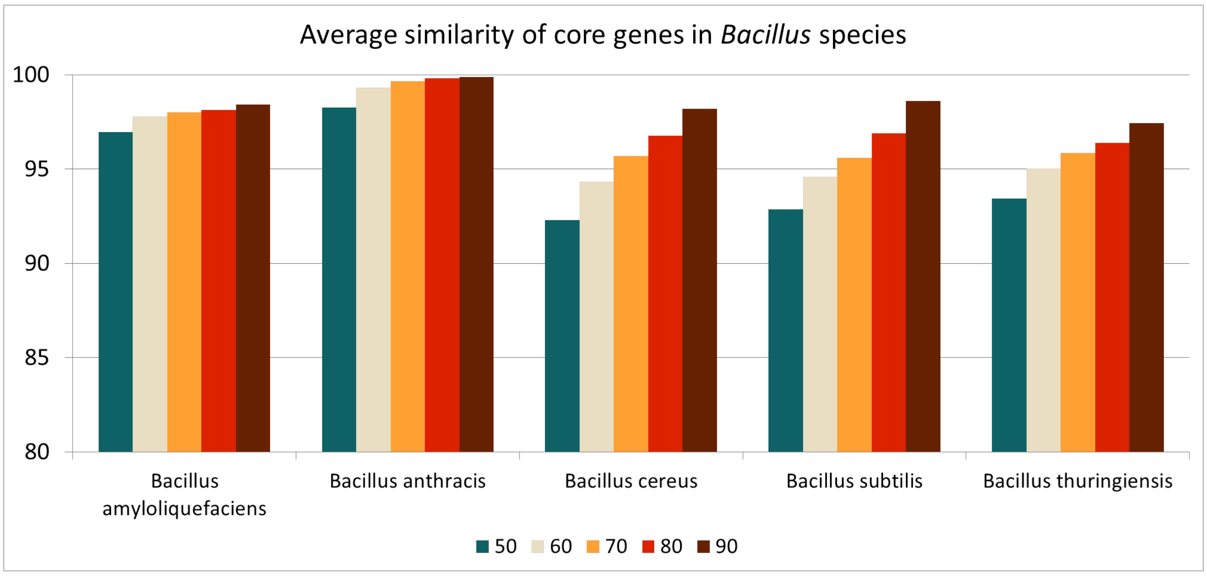

Supplementary Figure S1. Number of the core genes (top) and their average similarities in the orthologous gene cluster (bottom). Clustering thresholds ranges from 50% to 90% in different color.

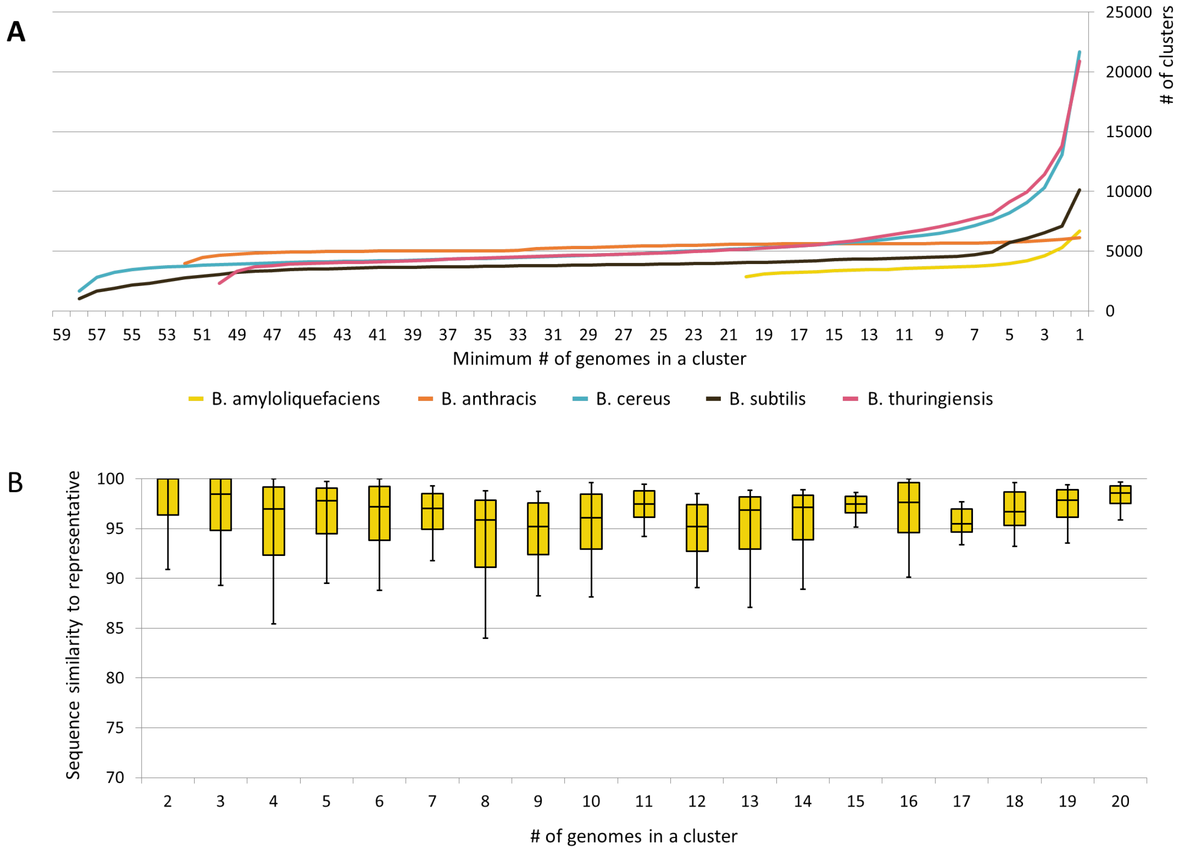

Supplementary Figure S2. Pan-genomes in five *Bacillus* species. (A) Total number of orthologous gene clusters with respect to the number of strains in a cluster. (B) Sequence similarity distribution of genes in each cluster of *B. amyloliquefaciens* with respect to the number of strains in a cluster.

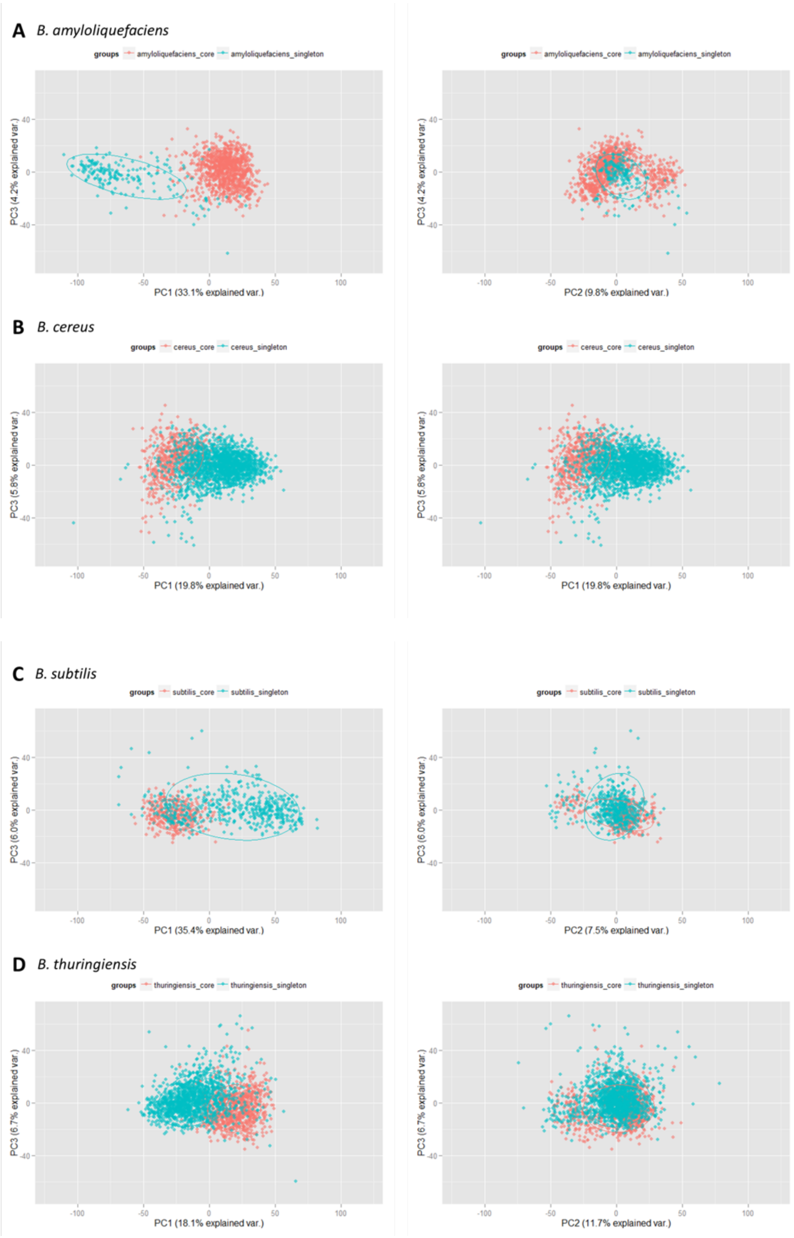

Supplementary Figure S3. Comparison of the genomic signatures (containing PC3) in core and strain-specific genes of *Bacillus* species.

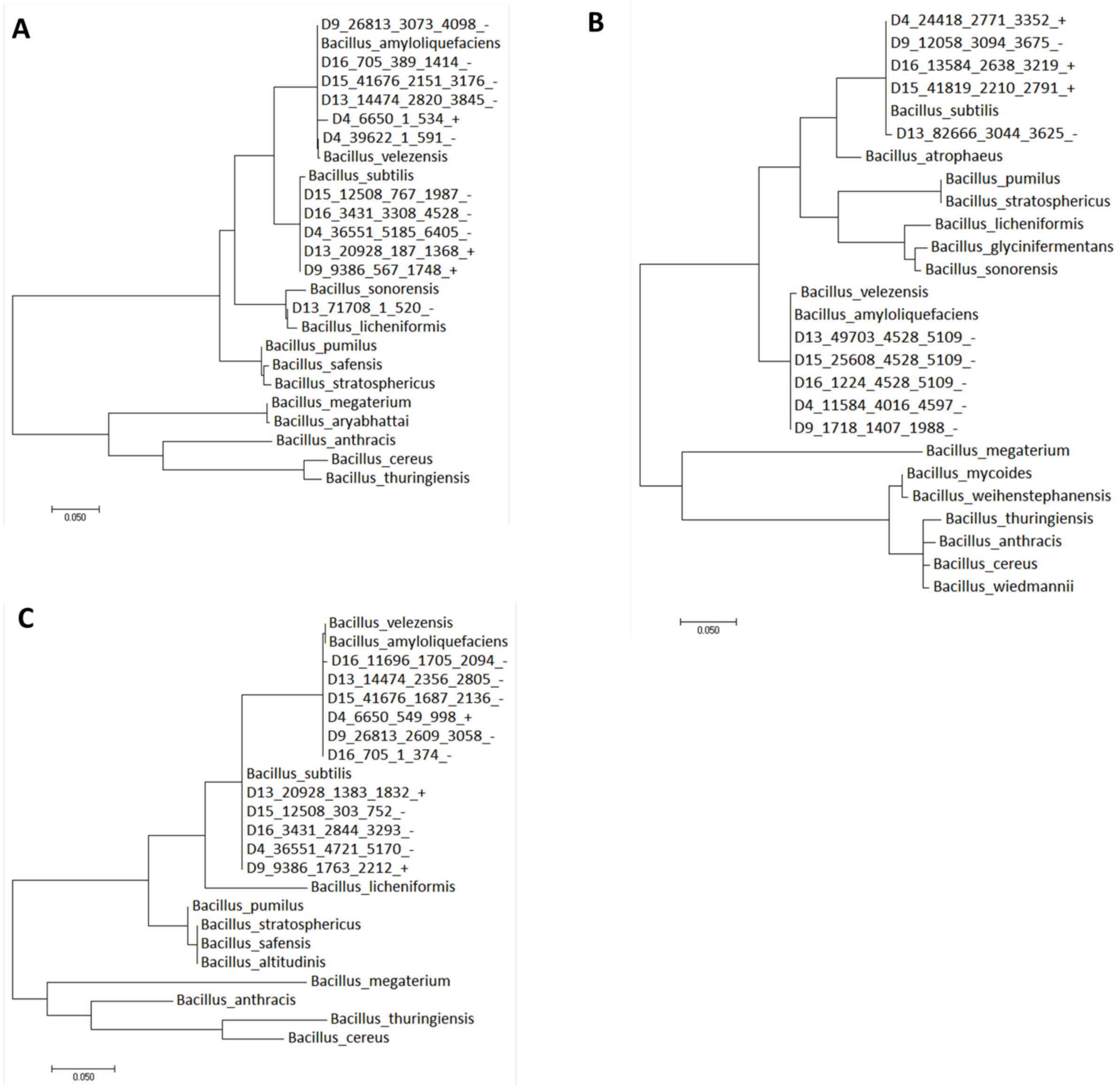

Supplementary Figure S4. Identification of *Bacillus* species in the *doenjang* microbiome. Phylogenetic trees of three *Bacillus* genes of (A) *pgs*A, (B) *pgs*B, (C) *pgs*C genes in five microbiomes D4, D9, D13, D15, and D16, along with the genes in the reference genomes.

Supplementary Table Legend

Supplementary Table S1. List of *Bacillus* genomes analyzed.

Supplementary Table S2. Average similarity of dispensable and core genes in each orthologous gene cluster among species for the different clustering thresholds.

Supplementary Table S3. Pan-genomes of the five *Bacillus* species with clustering of 70% threshold.

Supplementary Table S4. The ratio of the core genes clustered with the genes predicted in the *doenjang* microbiomes.

Supplementary Table S5. Microbiome profiling using MetaPhlAn (based on the marker genes) of the *doenjang* microbiome.

Supplementary Table S6. Sample information of the *doenjang* microbiome.

Supplementary Table S7. Statistical analysis of differential enhancement of COG functions in core and strain-specific genes.

Supplementary Table S1. List of *Bacillus* genomes analyzed.

strain	NCBI assembly	# of genes	genome size (Mb)	GC%
Bacillus amyloliquefaciens				
DSM 7	GCA_000196735.1	3922	3.9802	46.1
TA208	GCA_000195515.1	4089	3.93751	45.8
LL3	GCA_000204275.1	4228	4.00199	45.6938
XH7	GCA_000221645.1	4190	3.9392	45.8
IT-45	GCA_000242855.2	3860	3.93687	46.5876
Y2	GCA_000262385.1	4238	4.23862	45.9
CC178	GCA_000494835.1	3950	3.91683	46.5
LFB112	GCA_000508265.1	3859	3.94275	46.7
L-H15	GCA_000833005.1	3616	3.90597	46.7
KHG19	GCA_000835145.1	3675	3.95336	46.6
L-S60	GCA_000973485.1	3617	3.90302	46.7
MBE1283	GCA_001483885.1	3680	3.97993	46.4873
S499	GCA_001586105.1	3671	3.93593	46.5874
UMAF6639	GCA_001593765.1	3920	4.03464	46.3
UMAF6614	GCA_001593785.1	3894	4.00514	46.5
B15	GCA_001596755.1	3704	4.00675	46.5
RD7-7	GCA_001705195.1	3607	3.68821	46.3
Y14	GCA_001874385.1	3717	3.95716	46.4
LM2303	GCA_001889285.1	3739	3.98939	46.7
WS-8	GCA_001922005.1	3670	3.92979	46.5
B. anthracis				
Ames	GCA_000007845.1	5330	5.22729	35.4
Sterne	GCA_000008165.1	5287	5.22866	35.4
CDC 684	GCA_000021445.1	5896	5.50676	35.2646
A0248	GCA_000022865.1	5291	5.50393	35.2629
H9401	GCA_000258885.1	5791	5.49547	35.2644
A16	GCA_000512835.2	5530	5.50545	35.2629
SVA11	GCA_000583105.1	5741	5.48752	35.2625
HYU01	GCA_000725325.1	5317	5.49012	35.2642
2000031021	GCA_000742655.1	5509	5.33174	35.3573
Vollum	GCA_000742895.1	5732	5.50619	35.2646
Ames A0462	GCA_000830095.1	5529	5.50391	35.2646
PAK-1	GCA_000832425.1	5602	5.40338	35.3025
Vollum 1B	GCA_000832445.1	5732	5.50663	35.2646
K3	GCA_000832465.1	5695	5.50499	35.2648
Ohio ACB	GCA_000832505.1	5700	5.49834	35.2647
SK-102	GCA_000832565.1	5717	5.50566	35.2652

Pasteur	GCA_000832585.1	5472	5.2948	35.3571
BA1015	GCA_000832665.1	5687	5.49118	35.2643
BA1035	GCA_000832725.1	5746	5.48726	35.2624
RA3	GCA_000832745.1	5757	5.48987	35.2625
V770-NP-1R	GCA_000832785.1	5589	5.4104	35.3026
2002013094	GCA_000832965.1	5723	5.60108	35.2672
Canadian_bison	GCA_000833125.1	5706	5.50577	35.2647
Turkey32	GCA_000833275.1	5711	5.5053	35.2646
A1144	GCA_000875715.1	5525	5.47706	35.2621
Stendal	GCA_001543225.1	5536	5.50345	35.2629
Tangail-1	GCA_001654475.1	5524	5.5038	35.2629
Parent2	GCA_001683065.1	5295	5.22709	35.4
Parent1	GCA_001683095.1	5302	5.22866	35.4
PR01	GCA_001683135.1	5301	5.22866	35.4
PR02	GCA_001683155.1	5301	5.22866	35.4
PR05	GCA_001683175.1	5300	5.22866	35.4
PR06	GCA_001683195.1	5302	5.22866	35.4
PR07	GCA_001683215.1	5296	5.22709	35.4
PR08	GCA_001683235.1	5296	5.22709	35.4
PR09-1	GCA_001683255.1	5294	5.22706	35.4
PR09-4	GCA_001683275.1	5295	5.22709	35.4
PR10-4	GCA_001683295.1	5294	5.22708	35.4
Tyrol 4675	GCA_001936375.1	5693	5.50404	35.2647
SPV842_15	GCA_001990245.1	5634	5.41035	35.3026
delta Sterne	GCA_000742695.1	5479	5.22965	35.4
BFV	GCA_000742875.1	5702	5.50835	35.2705
A0157	GCA_000808075.1	5349	5.32224	35.4555
Pollino	GCA_000831505.1	5536	5.50389	35.2629
Larissa	GCA_001277955.1	5541	5.50329	35.263
8903-G	GCA_000558965.1	5500	5.50477	35.2647
9080-G	GCA_000558985.1	5605	5.50866	35.2631
52-G	GCA_000559005.1	5517	5.50444	35.2647
Smith 1013	GCA_000742315.1	5564	5.28699	35.2806
A16R	GCA_000512775.1	5276	5.40945	35.3026
Cvac02	GCA_000747335.1	5382	5.22717	35.4
Han	GCA_000747375.1	5513	5.22543	35.4
Bacillus cerues				
ATCC 14579	GCA_000007825.1	5255	5.42708	35.3076
ATCC 10987	GCA_000008005.1	5844	5.43265	35.5156
Q1	GCA_000013065.1	5502	5.50621	35.504
B4264	GCA_000021205.1	5398	5.41904	35.3

AH187	GCA_000021225.1	5783	5.59986	35.5213
G9842	GCA_000021305.1	5857	5.73682	35.0479
AH820	GCA_000021785.1	5810	5.58883	35.3086
03BB102	GCA_000022505.1	5606	5.44931	35.2945
CI	GCA_000143605.1	5558	5.48665	35.2707
NC7401	GCA_000283675.1	5761	5.55203	35.5372
FRI-35	GCA_000292415.1	5435	5.38232	35.4535
A1	GCA_000635895.2	5418	5.73461	35.135
03BB87	GCA_000789315.1	5788	5.71894	35.1791
D17	GCA_000832385.1	5651	5.59036	35.3171
FM1	GCA_000832525.1	5592	5.69776	35.2951
3a	GCA_000832765.1	5669	5.6423	35.2618
G9241	GCA_000832805.1	5683	5.72007	35.1812
ATCC 4342	GCA_000832845.1	5268	5.3063	35.3674
03BB108	GCA_000832865.1	6130	6.06873	34.9176
E33L	GCA_000833045.1	5838	5.84678	35.1686
S2-8	GCA_000835185.1	5674	5.64247	35.2618
FORC_005	GCA_000978375.1	5170	5.34962	35.3
NJ-W	GCA_001277915.1	5277	5.39851	35.1782
FORC_013	GCA_001518875.1	5683	5.67866	35.1948
CMCC P0021	GCA_001635915.1	5829	6.11289	34.9099
CMCC P0011	GCA_001635955.1	5828	6.09799	34.9092
HN001	GCA_001635995.1	5779	5.98083	34.921
FORC_024	GCA_001721145.1	5238	5.43012	35.3
FORC021	GCA_002000005.1	5418	5.37329	35.3101
BC-AK	GCA_002117465.1	5783	5.78633	35.3377
25	GCA_002117835.1	4931	4.82659	35.1
29	GCA_002117855.1	5107	4.99789	35.3
m1293	GCA_000003645.1	5282	5.26973	35.3501
AH1273	GCA_000003955.1	5798	5.7905	35.2502
ATCC 10876	GCA_000160895.1	5861	5.93902	34.8
BGSC 6E1	GCA_000160915.1	5687	5.73018	35
172560W	GCA_000160935.1	5641	5.69954	34.8
MM3	GCA_000160955.1	5553	5.54706	35.2
AH621	GCA_000160975.1	5667	5.67481	35.2
R309803	GCA_000160995.1	5587	5.58625	35.4
m1550	GCA_000161035.1	5245	5.24649	35.1
BDRD-ST24	GCA_000161055.1	5417	5.43617	35.1
BDRD-ST196	GCA_000161095.1	5549	5.57657	35.2
BDRD-Cer4	GCA_000161115.1	5372	5.39745	35.1
Rock1-3	GCA_000161155.1	5815	5.85985	34.9

Rock1-15	GCA_000161175.1	5787	5.76628	34.9
Rock3-29	GCA_000161215.1	5868	5.87811	34.9
Rock3-42	GCA_000161235.1	5313	5.2029	35.2
Rock3-44	GCA_000161255.1	4750	4.9998	36.7505
Rock4-2	GCA_000161275.1	5715	5.76999	34.9
F65185	GCA_000161315.1	6027	6.13318	34.7
AH603	GCA_000161335.1	5799	5.79945	35.1
AH676	GCA_000161355.1	5612	5.59475	35
AH1271	GCA_000161375.1	5666	5.6567	35.3
AH1272	GCA_000161395.1	5810	5.78954	35.2
FT9	GCA_000724585.1	4278	5.22366	35.5
BDRD-ST26	GCA_000161075.1	5778	5.56803	35.2
Rock3-28	GCA_000161195.1	5728	6.0415	35
Bacillus subtilis				
W23	GCA_000146565.1	4063	4.02768	43.9
BSn5	GCA_000186745.1	4145	4.0936	43.8
BEST195	GCA_000209795.2	4477	4.11122	43.4962
TU-B-10	GCA_000227465.1	4297	4.20722	43.8
RO-NN-1	GCA_000227485.1	4101	4.01195	43.9
QB928	GCA_000293765.1	4034	4.14684	43.6
BSP1	GCA_000321395.1	3847	4.04375	43.9
XF-1	GCA_000338735.1	3853	4.06119	43.9
6051-HGW	GCA_000344745.1	4188	4.21561	43.5
BAB-1	GCA_000349795.1	4003	4.02194	43.9
PY79	GCA_000497485.1	4138	4.03346	43.8
BEST7003	GCA_000523045.1	4011	4.04304	43.9
JH642 substr. AG174	GCA_000699465.1	4227	4.18837	43.5
AG1839	GCA_000699525.1	4231	4.19364	43.5
OH 131.1	GCA_000706705.1	3885	4.03916	43.8
TO-A	GCA_000737405.1	4083	4.05749	43.8
ATCC 13952	GCA_000772125.1	3852	3.87628	45.8
ATCC 19217	GCA_000772165.1	3677	3.9599	46.4
Bs-916	GCA_000772205.1	3741	3.98167	46.5
SG6	GCA_000782835.1	4017	4.07967	43.8
168	GCA_000789275.1	4307	4.21562	43.5
PS832	GCA_000789295.1	4299	4.21537	43.5
3NA	GCA_000827065.1	4277	4.1951	43.6
BS49	GCA_000953615.1	4248	4.25165	43.5
KCTC 1028	GCA_000971925.1	4303	4.21563	43.5
HJ5	GCA_000973605.1	3917	4.01293	43.8
UD1022	GCA_001015095.1	3933	4.02533	43.9

TO-A JPC	GCA_001037985.1	4231	4.09071	43.8
BSD-2	GCA_001465815.1	3951	4.03084	43.9
DE111	GCA_001534785.1	4066	4.14389	43.9
CU1050	GCA_001541905.1	4064	4.05628	43.9
CGMCC 2108	GCA_001565875.1	4182	4.19375	43.407
ATCC 49760	GCA_001597265.1	3950	4.17548	43.2
SZMC 6179J	GCA_001604995.1	4276	4.1952	43.6
delta6	GCA_001660525.1	3928	3.87692	43.9
KCTC 3135	GCA_001697265.1	4301	4.21134	43.5
168G	GCA_001703495.1	4297	4.21481	43.5
HJ0-6	GCA_001704095.1	4245	4.23512	43.5
BS16045	GCA_001720505.1	4192	4.16512	43.6
BS38	GCA_001746575.1	4159	4.01042	43.6
HRBS-10TDI13	GCA_001747445.1	4135	4.19542	43.2906
VV2	GCA_001808235.1	3921	4.01382	43.9
J-5	GCA_001889385.1	3917	4.1179	46.1
MJ01	GCA_001889625.1	4027	4.10829	43.9
KH2	GCA_001890405.1	4222	4.21243	43.4049
29R7-12	GCA_001902555.1	4187	4.20405	43.4008
NCIB 3610	GCA_002055965.1	4335	4.29982	43.3355
GQJK2	GCA_002072735.1	3976	4.07296	43.8
Bs-115	GCA_002096095.1	4037	4.14259	43.5
B-1	GCA_000769515.1	3706	3.94145	46.5
QB5412	GCA_001750745.1	4300	4.21795	43.5
NRS 231	GCA_000816805.1 (contaminated)	3968	4.02767	43.9
T30	GCA_000959025.1 (contaminated)	3896	4.03173	43.9
D12-5	GCA_001596535.1	3746	4.14264	43.6
IIG-Bs27-47-24	GCA_001698485.1	2902	2.90231	44.3
PG10	GCA_001698505.1	2756	2.7593	44.2
PS38	GCA_001698525.1	2691	2.68033	44.1
QB5413	GCA_001750765.1	5099	4.21796	42.7
Bacillus thuringiensis				
97-27	GCA_000833085.1	5327	5.31269	35.3595
Al Hakam	GCA_000015065.1	4798	5.31303	35.4084
ATCC 10792	GCA_000161615.1	6243	6.26014	34.8
Bc601	GCA_001618665.1	5931	6.11129	35.0942
BGSC 4AA1	GCA_000940785.1	6011	6.1799	35.0858
BGSC 4AJ1	GCA_000161595.1	6490	6.48902	34.7
BGSC 4AW1	GCA_000161635.1	5546	5.48884	35.1
BGSC 4BA1	GCA_000161655.1	6053	6.03148	34.9

BGSC 4BD1	GCA_000161675.1	6019	6.2312	34.6501
BGSC 4C1	GCA_001640965.1	5626	5.81812	35.2064
BGSC 4CC1	GCA_000161695.1	5944	6.0026	34.9
BGSC 4Y1	GCA_000161475.1	5732	5.62591	34.9
BMB171	GCA_000092165.1	5349	5.64305	35.1891
Bt18247	GCA_001721165.1	6210	6.1382	35.1552
Bt185	GCA_001595725.1	5894	6.3905	34.8215
Bt407	GCA_000306745.1	6402	6.13434	35.0166
CT-43	GCA_000193355.1	6206	6.15115	35.1212
CTC	GCA_001455345.1	5307	5.35293	35.3976
HD 1	GCA_000835235.1	6957	6.85947	34.8925
HD-1	GCA_000717535.1	6782	6.76659	34.9182
HD1002	GCA_000835025.1	6665	6.5727	35.0677
HD1011	GCA_000832485.1	6088	6.09337	35.1468
HD12	GCA_001598095.1	6192	6.49046	35.1836
HD-29	GCA_000803665.1	6522	6.74223	34.9557
HD521	GCA_001183785.1	6172	6.19845	34.9666
HD571	GCA_000832825.1	5339	5.31218	35.4084
HD682	GCA_000832925.1	5292	5.29139	35.4811
HD73	GCA_000338755.1	6194	5.90857	35.1904
HD-771	GCA_000292455.1	6569	6.43837	35.0432
HD-789	GCA_000292705.1	6462	6.33463	35.1765
HS18-1	GCA_001182785.1	6127	6.40346	34.9753
IBL 200	GCA_000161715.1	6693	6.73179	34.5
IBL 4222	GCA_000161735.1	6658	6.61243	34.8501
IS5056	GCA_000341665.1	6922	6.77159	34.905
KNU-07	GCA_001692675.1	5743	6.15274	34.8926
L-7601	GCA_002025105.1	6179	6.30377	35.052
MC28	GCA_000300475.1	6557	6.69453	34.9245
MYBT18246	GCA_001685565.1	7089	6.75249	35.3991
Pasteur Institute Standard strain	GCA_001548175.1	7044	6.87059	35.0603
ST7	GCA_001675515.1	5675	6.28612	35.1242
T01001	GCA_000161515.1	6323	6.32312	34.8
T03a001	GCA_000161575.1	5556	5.52757	35
T04001	GCA_000161535.1	6583	6.10775	34.6
T13001	GCA_000161555.1	6028	6.03751	35
XL6	GCA_000774075.2	5534	5.70184	34.6308
YBT-020	GCA_000190515.1	5782	5.68238	35.3815
YBT-1518	GCA_000497525.2	6738	6.67292	35.2915
YBT-1520	GCA_000747545.1	6816	6.52041	34.9377
YC-10	GCA_001017635.1	6508	6.78414	34.9011

YWC2-8	GCA_001420855.1	6193	6.22794	35.0998

Supplementary Table S2. Average similarity of dispensable and core genes in each orthologous gene cluster among species for the different clustering thresholds.

	Dispensable genes			Core genes		
Similarity (%) /length aligned (bp)	min	avg	max	min	avg	max
50/50	86.66	91.61	98.57	92.30	94.76	98.27
60/60	91.05	94.26	99.39	94.33	96.21	99.32
70/70	92.87	95.44	99.53	95.60	96.97	99.67
80/80	94.51	96.57	99.66	96.41	97.61	99.84
90/90	96.84	97.93	99.77	97.45	98.52	99.90

Supplementary Table S3. Pan-genomes of the five *Bacillus* species with clustering of 70% threshold.

# of genome	*Bacillus amyloliquefaciens*		*Bacillus anthracis*		*Bacillus cereus*		*Bacillus subtilis*		*Bacillus thuringiensis*	
	# of cluster	average similarity	# of clusters	average similarity	# of clusters	average similarity	# of clusters	average similarity	# of clusters	average similarity
1	1364		156		8617		3013		7087	
2	703	96.82	82	99.98	2768	94.82	551	93.91	2364	94.85
3	384	95.80	113	99.43	1222	93.05	463	95.51	1500	95.02
4	235	94.73	48	99.79	883	92.55	378	95.38	812	93.34
5	146	96.11	41	99.74	582	92.50	772	95.69	997	96.31
6	73	95.55	18	99.24	446	92.26	234	93.28	403	92.88
7	66	95.38	16	99.03	389	92.09	143	96.57	359	93.42
8	42	93.86	12	99.71	260	91.59	59	92.56	322	93.29
9	47	94.37	12	99.84	190	91.60	47	91.58	272	93.94
10	62	94.73	7	99.65	169	90.87	46	91.81	230	93.19
11	58	96.56	5	99.53	150	91.31	20	93.68	246	93.23
12	28	94.50	5	99.62	135	91.01	53	93.02	197	93.01
13	29	95.59	6	99.67	129	91.09	23	91.66	227	94.08
14	47	95.52	6	100.00	107	92.25	51	96.84	161	94.27
15	96	96.87	3	99.93	96	91.75	60	97.70	166	95.42
16	39	96.26	4	99.94	97	90.88	45	96.20	94	92.23
17	47	95.31	4	99.63	69	92.36	55	95.67	91	91.06
18	92	96.37	9	100.00	72	91.40	39	96.51	101	91.12
19	228	97.11	4	100.00	71	90.42	31	93.96	75	92.53
20	2870	98.03	12	98.30	70	92.21	33	95.38	86	91.56

21	38	99.31	72	90.46	32	96.80	65	90.98
22	30	99.62	56	91.33	23	95.32	59	91.56
23	28	99.19	72	91.76	18	97.35	62	91.61
24	30	99.58	74	91.07	19	92.68	60	92.44
25	25	99.59	69	90.69	23	94.85	58	91.13
26	43	99.57	55	91.45	17	94.18	43	91.93
27	36	99.65	58	89.88	14	94.69	45	90.77
28	35	99.29	44	89.97	22	94.80	35	91.03
29	26	99.61	51	91.87	21	96.18	31	91.27
30	44	99.80	40	89.41	16	95.45	39	91.69
31	47	98.99	41	89.59	16	96.30	27	92.61
32	143	99.67	36	91.25	17	95.92	39	92.48
33	11	99.73	41	91.42	20	94.91	38	91.46
34	7	99.78	45	89.15	16	91.84	49	92.33
35	4	99.77	37	92.96	16	95.73	58	92.30
36	5	93.64	36	90.16	13	93.24	60	91.97
37	4	99.25	50	89.38	13	96.71	67	93.70
38	3	99.72	32	90.07	20	95.55	55	93.16
39	3	99.96	39	91.36	14	91.33	42	92.63
40	10	99.96	24	89.51	30	91.80	46	92.61
41	4	99.74	27	90.59	30	93.43	40	90.41
42	8	99.85	27	91.66	40	94.03	35	93.36
43	15	98.66	22	90.85	40	93.27	33	91.05
44	24	99.76	26	89.94	26	92.88	44	93.39
45	37	98.93	39	92.30	43	92.02	62	92.86
46	49	99.13	37	91.83	53	93.98	103	92.66
47	46	99.63	39	89.81	78	94.91	121	93.88

48			84	99.41	50	91.42	95	93.98	335	94.00
49			97	99.28	46	90.08	149	93.87	1042	95.34
50			180	99.46	57	89.47	168	94.26	2299	95.85
51			484	99.70	67	91.66	135	93.34		
52			3972	99.67	70	91.36	237	93.36		
53					78	91.14	210	92.72		
54					124	92.69	149	93.54		
55					234	92.57	287	94.47		
56					445	92.61	213	94.23		
57					1137	94.01	645	94.92		
58					1656	95.70	1022	95.60		
total	6656	97.14	6135	99.62	21675	93.23	10116	94.71	20882	94.46

[+] # of clusters summed. [*] the average similarity of the entire clusters.

Supplementary Table S4. The ratio of the core genes clustered with the genes predicted in the *doenjang* microbiomes.

Similarity (%)	sample ID	*B. amyloliquefaciens*	*B. subtilis*	*B. thuringiensis*	*B. cereus*	*B. anthracis*
70	D13	99.44	99.80	21.01	27.66	18.81
	D15	99.13	99.41	16.79	23.85	14.85
	D16	99.13	99.71	20.14	26.93	17.70
	D4	99.30	99.80	21.44	28.38	18.55
	D9	99.44	99.80	23.75	30.43	20.87
	Avg.	99.29	99.71	20.63	27.45	18.16
90	D13	98.36	97.26	1.21	0.52	0.63
	D15	98.54	96.38	1.03	0.30	0.45
	D16	97.80	98.73	1.33	0.52	0.70
	D4	97.70	97.70	0.65	1.57	0.98
	D9	98.68	98.68	2.29	1.22	1.51
	Avg.	98.22	97.77	1.30	0.83	0.86

Supplementary Table S5. Microbiome profiling using MetaPhlAn (based on the marker genes) of the *doenjang* microbiome.

ID	D13	D15	D16	D4	D9
s__Bacillus_amyloliquefaciens	7.99[+]	15.35	23.52	17.00	13.27
s__Bacillus_coagulans	8.84	0	0	0	0
s__Bacillus_licheniformis	10.33	11.29	13.08	17.59	19.86
s__Bacillus_smithii	6.27	0	0	0	0
s__Bacillus_sonorensis	9.78	3.70	0	17.93	13.17
s__Bacillus_sp_BT1B_CT2	7.77	0	0	0	0
s__Bacillus_subtilis	8.33	12.47	19.79	13.78	12.66
s__Bacillus_vallismortis	0	0.63	0	0	0
s__Bacillus_phage_phiNIT1	0	0	27.14	0	0

[+] the proportion is represented as a percentage.

Supplementary Table S6. Sample information of the *doenjang* microbiome.

ID	D13	D15	D16	D4	D9
Read count	82,190,136	72,332,136	76,172,238	82,045,316	94,990,796
Base pairs (Gbps)	8.3	7.3	7.7	8.2	9.6

Supplementary Table S7. Statistical analysis of differential enhancement of COG functions in core and strain-specific genes.

	Class	Symbol	Enhanced group	p-value
Cellular processing and signaling	Cell cycle control, cell division, chromosome partitioning	D	Core	0.0343
	Cell wall/membrane/envelope biogenesis	M		0.6072
	Cell motility	N		0.8950
	Posttranslational modification, protein turnover, chaperones	O	Core	0.0095
	Signal transduction mechanisms	T		0.2068
	Intracellular trafficking, secretion, and vesicular transport	U		0.6513
	Defense mechanisms	V	Strain	0.0281
	Extracellular structures	W		0.2258
	Cytoskeleton	Z	Core	0.0270
Information storage and processing	RNA processing and modification	A		0.2050
	Chromatin structure and dynamics	B		0.3832
	Translation, ribosomal structure and biogenesis	J	Core	0.0001
	Transcription	K	Strain	0.0016
	Replication, recombination and repair	L	Strain	1.E-05
Metabolism	Energy production and conversion	C	Core	6.E-05
	Amino acid transport and metabolism	E	Core	0.0013
	Nucleotide transport and metabolism	F		0.5030
	Carbohydrate transport and metabolism	G		0.4203
	Coenzyme transport and metabolism	H	Core	2.E-05
	Lipid transport and metabolism	I		0.0594
	Inorganic ion transport and metabolism	P	Core	6.E-06
	Secondary metabolites biosynthesis, transport and catabolism	Q	Strain	0.0004
Poorly characterized	General function prediction only	R		0.2998
	Function unknown	S		0.1274
Mobilome	Mobileome	X	Strain	0.0128
